# Entropy, complexity, and spatial information

**DOI:** 10.1007/s10109-014-0202-2

**Published:** 2014-09-24

**Authors:** Michael Batty, Robin Morphet, Paolo Masucci, Kiril Stanilov

**Affiliations:** 1 Centre for Advanced Spatial Analysis (CASA), University College London (UCL), 90 Tottenham Court Road, London, W1N 6TR UK; 2 Department of Architecture, The Martin Centre for Architectural and Urban Studies, 1-5 Scroope Terrace, Trumpington Street, Cambridge, CB2 1PX UK

**Keywords:** Information, Entropy, Density, Spatial complexity, London population, London street system, C46, R12, R14, R40, R52

## Abstract

We pose the central problem of defining a measure of complexity, specifically for spatial systems in general, city systems in particular. The measures we adopt are based on Shannon’s (in Bell Syst Tech J 27:379–423, 623–656, 1948) definition of information. We introduce this measure and argue that increasing information is equivalent to increasing complexity, and we show that for spatial distributions, this involves a trade-off between the density of the distribution and the number of events that characterize it; as cities get bigger and are characterized by more events—more places or locations, information increases, all other things being equal. But sometimes the distribution changes at a faster rate than the number of events and thus information can decrease even if a city grows. We develop these ideas using various information measures. We first demonstrate their applicability to various distributions of population in London over the last 100 years, then to a wider region of London which is divided into bands of zones at increasing distances from the core, and finally to the evolution of the street system that characterizes the built-up area of London from 1786 to the present day. We conclude by arguing that we need to relate these measures to other measures of complexity, to choose a wider array of examples, and to extend the analysis to two-dimensional spatial systems.

## Introduction

Complexity by its very nature is an impossible term to define. Anderson (1972), whose seminal paper ‘More is Different’ written 40 years ago, poses this dilemma in the very title of his paper. Complex systems defy definition in that they are intrinsically unpredictable and depend on innovations that occur in the future and cannot be forecast. Any definition of complexity should take into account its fundamentally anthropomorphic nature. When we seek to describe a system, we implicitly define an object—the system, and a subject—the observer or modeller. The object or rather its representation is analysed into parts whose interactions are to be studied and modelled. The definition of object and model is a matter of choice, and thus, its complexity is dependent on this choice and the state of knowledge associated with the observer (Badii and Politi 1997). More recently, Anderson (2011) has followed his earlier speculation saying that complexity “… is the search for general concepts, principles and methods for dealing with systems which are so large and intricate that they show autonomous behaviour which is not just reducible to the properties of the parts of which they are made” (pp. 364–365).

There is a sense in all of this that as systems change, they vary in their complexity and that as cities grow for example and their populations get richer, they get ‘more complex’. This poses the question as to whether it is possible to present a measure of complexity sufficiently general to pick up change in scale and size and of course diversity (which is a key idea in complexity) that unequivocally show how cities change in these terms. We would expect, for example, that as cities get larger, they get more complex although this is by no means certain. There are examples in history of empires that have grown while their cities have collapsed and their complexity has probably fallen. Indeed, when populations have fallen through dispersion from the core, or where epidemics have led to such reductions, complexity has probably decreased and thus we need a measure that depends not only on numbers, scale and size, but also on the way that their populations are distributed spatially.

In this paper, we will begin with the simplest and most general of measures, arguing that the generic formula for information due to Shannon (1948) is a good starting point. Shannon’s measure links fundamentally to the relations between complexity, Ashby’s variety, entropy, and cybernetics which has been cogently argued by Casti (1996) and more recently set in its historical context by Gershenson et al. (2013). The concept of entropy is also familiar in spatial analysis from the development of entropy-maximizing spatial interaction models (Wilson 1970) although our treatment of these origins is peripheral to the argument here. As the number of events increases, information increases according to this formula, but at the same time the distribution of events—the way they are ordered—also affects the measure. When something is entirely ordered, hence completely predictable, it is no longer complex, and we would expect the measure to be low relative to a situation of extreme unpredictability. However, we can also argue that something that is entirely ordered has great complexity in that to simply hold this order together in far-from-equilibrium systems, considerable intricacy in structure and relations is required. This is in contrast to a situation where events are completely unpredictable, hence in one sense quite disordered and possibly of low complexity in that random forces dominate.

This dilemma of interpretation is central to our argument for it suggests that there are many kinds of complexity dependent upon the perspective one is taking. Here, highly ordered events with great predictability are regarded as much less complex than those where we are unable to predict their order, and it is in this sense that we say these sets of disordered events are more complex. The Shannon information measure relates to semantic information through the variables used to define its underlying probability distribution; in this case, the variables are area and population and this defines the analytic perspective that is at a higher level of abstraction than the application of semantic information to urban artefacts as described by Haken and Portugali (2003). The measure, in its statistical mechanical context as argued by Jaynes (1965), is anthropomorphic in the sense discussed above.

We will see that the measures we propose meet these criteria, but our analysis is elementary at this stage. It might be argued that population and size in terms of the way populations are defined are far from the kinds of complexity that the complexity sciences are currently focused upon. Nevertheless, we see this as a starting point, picking up on a long tradition of measuring and using information in spatial analysis while at the same time, providing new directions with respect to how complex systems such as cities are evolving. In this sense, our focus is on the dynamics of cities because dynamics raises the question of increasing (or decreasing) complexity (Batty 2010). In essence, although Shannon’s measure of information pertains in general to closed systems, we admit increasing complexity into our system by handling growth through additional numbers of events (locations), thus letting the measure vary with respect to size and scale as well as the intrinsic distribution of the events. In conventional applications, the number of events is regarded as fixed but here we will relax these for as cities grow, the number of locations that define them increases. In a time when the world’s population will be largely living in cities by the end of this century, our concern for this kind of measurement would appear appropriate.

We will first present standard information measures, defining information for the one-dimensional case, a city strung out on a line for example or a series of events whose spatial relations and interactions are one dimensional. We then examine relative information, introducing the concept of partitioning information or entropy into two key components—a spatial entropy that largely deals with the distribution of information (Batty 1974; Esmer 2011) and an information density that deals with size. These measures illustrate how the various components of distribution, numbers of events and their density trade-off against one another as the system changes in size and scale. In this sense, we are able to show that cities can grow in size but change in the distribution of their populations, leading to either an increase or a decrease in their complexity. We will then explore these notions for population change in Greater London using several different data based on different measures of population and area at different temporal cross-sections.

First, we will look at population change in the 33 London boroughs over the last 100 years from 1901 to 2001. The area units—the boroughs—do not change in size or shape (because we have normalised the areas to current 2001 boundaries), and thus, the analysis focuses on defining information that reflects changes in population density, not the number of areal units. Then, for the same data, we will explore the pattern at single cross-section in 2001 with respect to how information changes as we keep the population fixed while varying the number of zones from 2 to 33. In this way, we examine how complexity changes with the number of events, searching for points at which the complexity of the metropolis falls even when the number of zones increases. Our example makes the point, but as this is somewhat unrealistic, we then move to examine a much larger region centred on London based on 1,767 zones which comprises the entire metropolitan area at 2001. We change the numbers of zones which we arrange in 11 zonal bands at different distances from the centre, and with this wide range of variation in population density and numbers of zones, we search for distinct changes in complexity in spatial terms. To evaluate the trade-off between population density and numbers of zones, we then use a very different data set which measures the number of street intersections on a regular grid, where the data are equivalent to street density. These data are available for nine time periods from 1786 until 2010. As the number of zones increases through time, we use a threshold to determine densities of significance which increase through time as the city grows and consolidates its form. In this last example, we can see the trade-off between the density and the number of events. We then conclude with suggestions as to how this kind of analysis might be taken forward for interactions between locations, moving these ideas from the one-dimensional to the two-dimensional realm which will constitute the basis of future work.

## Defining information

In spatial analysis, the most general way of defining a continuously varying distribution is in terms of frequencies or probabilities where the probability *p*_*i*_ of the occurrence of an event *i* varies with respect to some attribute of the system, often size *x*_*i*_. Probabilities vary between 0 and 1 and are normalized so that they sum to 1, that is

1 $$ \sum\limits_{i = 1}^{n} {p_{i} = 1} , $$

where *n* is the range of probable events. It is this conservation of events that restricts the measure to a closed system, and of course, if the number of events changes, then this implies that the system’s relative closure changes. If an event occurs, then the information that is gained varies inversely with the size of the probability. When the probability of the event is very small and the event occurs, then the information gained is high in comparison with a situation where the probability is very likely. In the extreme case where the probability of the event occurring is 1, then no information is gained. In general, then we assume that information gained varies as 1/*p*_*i*_ but the best form of this function needs to be determined with respect to other criteria.

To formalize these ideas, let us assume that there are two events *n* = 2 with probabilities *p*_1_ and *p*_2_ where *p*_1_ + *p*_2_ = 1. If the two events are independent and then occur together with probability *p*_1_ *p*_2_, we would expect that the information gained would be proportional to 1/*p*_1_ *p*_2_, but we would also expect the information to be additive, that is, the information gained to be 1/*p*_1_ + 1/*p*_2_ because it does not matter in which order truly independent events happen. But this is not equal to 1/*p*_1_ *p*_2_ so what we require is a functional form for information gained that ensures that

2 $$ f\left( {\frac{1}{{p_{1} p_{2} }}} \right) = f\left( {\frac{1}{{p_{1} }}} \right) + f\left( {\frac{1}{{p_{2} }}} \right). $$

The only function satisfying Eq. (2) is log(1/*p*) which when substituted in Eq. (2) yields the equality. However, this equation does not give a value for the overall information for the two events. To generate this, we need to compute the expected value *H*(2) which for the two-event example is

3 $$ H(2) = - p_{1} \,\log p_{1} - p_{2} \,\log p_{2} , $$

and for a system of *n* events, Eq. (3) generalizes to

4 $$ H = H\left( n \right) = - \sum\limits_{i} {p_{i}  \,{ \log } p_{i} } . $$

We can now drop explicit reference to the number of events *n*, and note that *H* is the standard formula for information derived by Shannon (1948), equivalent to the Boltzmann–Gibbs formula for entropy.

There are two other properties of Shannon’s formula that are of essential interest to our subsequent analysis of spatial information. It is easy to show that Eq. (4) varies from a minimum value of zero to a maximum value of log(*n*). In these cases, the distribution of probabilities has a unique form. When *H* = 0, then one event dominates, that is, *p*_*k*_ = 1, ∀*p*_*i*_ = 0, *i* ≠ *k* while when *H* = log(*n*), then *p*_*i*_ = 1/*n*, ∀*i*. If the distribution were, say, population in the zones of a city, if everyone is located in one zone, the information gained is zero when an event occurs.^1^ When the distribution of population is uniformly spread in every zone of the city, and an event occurs, this gives maximum information. The former example might be for a high-density, highly ordered city and the latter for an entirely spread out city where accessibility was of no importance. Many different configurations exist in between. There is thus a trade-off between the spread of the probability distribution and the number of events. One of the main goals of this paper is to examine this trade-off for we believe that as cities get more complex and bigger, they are characterized by more and more events, and in this sense, we can treat cities as being open. But at the same time, they may change in shape in that they may suburbanize or densify and this may lead to an increase in information or to a decrease. The trade-off between density and the number of events is thus the crucial issue in using the *H* measure as an index of complexity. To make this clear, we will now examine the simplest example of a two- and then three-event/zone system that enables us to demonstrate the feasibility of such trade-offs.

**Fig. 1:**
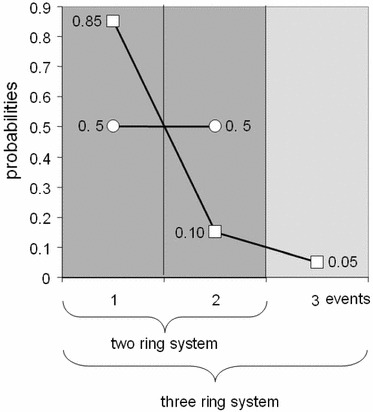
The two-event and three-event systems

Imagine a city that is divided into two rings around a central business district (CBD). The population is evenly distributed in each ring, and the probability of locating in the two rings is [0.5, 0.5]. Let us say there is a period of sustained growth and redistribution, sufficient for the city to change its evenly spread profile to a much more concentrated form around the CBD given now by three rings where the city has grown outwards, as [0.85, 0.1, 0.05]. This is a perfectly feasible shift in distribution from, for example, a walking city of the pre-industrial era to an industrial city where land use and activities are concentrated in the centre. Note that we have said nothing about the size of the population for this is not part of our measure of information, nor have we said anything about the size of the zones or rings, and this will be an important part of analysis in later sections. Now the entropy of the two-ring city is *H* = log(2) = 0.301, and the entropy of the three-ring city is *H* = 0.225, substantially less than the previous form despite the fact that the city has grown in terms of the number of rings used to describe its growth. Had the city remained as a uniform spread, then the distribution would have been [0.33, 0.33, 0.33] and the entropy *H* would have increased to 0.477. Instead of a 25 % decrease in information, the even spread would have led to a 58 % increase. This in essence is the trade-off between density and number of events that we are seeking to understand and formalize in the rest of this paper. In short, we assume that as cities grow, their complexity in terms of information increases but this is purely based on the number of their zones and there may be strong ordering effects that discount this increase. This change is shown in Fig. 1 for the two systems in question.

**Fig. 2:**
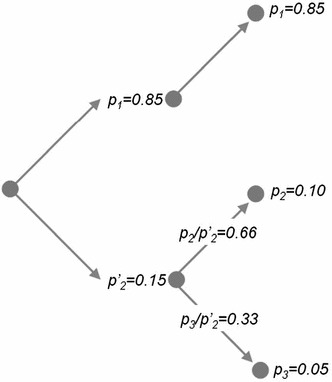
Hierarchical aggregation–decomposition in the three-event system

The second property of the entropy involves the additive independence of the events that describe it. Imagine now that our three-event system with probabilities [0.85, 0.1, 0.05] is aggregated back to two events *p*_1_ and $p_{2}^{\prime }$ with the second and third events $p_{ 2} + p_{ 3} = p_{2}^{\prime } = 0. 10 + 0.0 5 = 0. 1 5$ of the original system forming the new event. Our new two-event system is now [0.85, 0.15]. If we now work out the relative probabilities of the two old events that comprise the new event, then $p_{2} /p_{2}^{{\prime }} = 0. 1/0. 1 5 = 0. 6 6 6$ and $p_{3} /p_{2}^{{\prime }} = 0. 0 5/0. 1 5 = 0. 3 3 3$. It is easy to see that the original probabilities for the second and third events can be recovered by multiplying the probability 0.15 by the relative probabilities giving *p*_2_ = 0.15 × 0.666 = 0.1 and *p*_3_ = 0.15 × 0.333 = 0.05. The new entropy is the sum of the new aggregated two-event entropy $- p_{1} \log \rlap{-} p_{1} - p_{2}^{{\prime }} \log p_{2}^{{\prime }}$ and a weighted sum of $- p_{2}^{{\prime }} \left[ {\left( {p_{ 2} /p_{2}^{{\prime }} } \right) { \log } \left( {p_{ 2} /p_{2}^{{\prime }} } \right) + \left( {p_{ 3} /p_{2}^{{\prime }} } \right) { \log } \left( {p_{ 3} /p_{2}^{{\prime }} } \right)} \right]$ which is the subdivided entropy term. Noting that the overall entropy of the three-event system is 0.225, this subdivides into 0.184, the aggregated entropy, and 0.041, the disaggregated term. The hierarchical subdivision is shown in Fig. 2. This can be generalized to *n* events grouped into *m* sets, and we can now write *H* as

5 $$ \left. \begin{gathered} H = - \sum\limits_{k}^{m} {P_{k} \,\log P_{k} } - \sum\limits_{k}^{m} {P_{k} \sum\limits_{{i \in\Omega _{k} }}^{{}} {\frac{{p_{i} }}{{P_{k} }}\log } } \frac{{p_{i} }}{{P_{k} }} \hfill \\ P_{k} = \sum\limits_{{i \in\Omega _{i} }} {p_{i} } \quad {\text{and}}\quad \sum\limits_{k}^{m} {P_{k} = 1} \hfill \\ \end{gathered} \right\}, $$

where there are *m* sets Ω_*k*_ which contain the aggregate probabilities. Because of the recursion implied in Eq. (5), it is possible to decompose the probabilities into a tree-like hierarchy as shown in Fig. 2, and in this way, the information is associated with different levels of the spatial hierarchy. This has already been explored by Batty (1976).

## Measuring relative information

### The information difference

Information as defined in the *H* measure contains an implicit assumption that when the formula is used to compute information, this information is relative to some baseline assumption. In the case of *H*, this is the basic probability distribution that acts as a kind of prior since information is relative to what actually occurs. Of course, this event sequence is unknown; hence, the measure is quite general, but other measures are more specific in that they compare the actual to various assumed distributions. This is first accomplished by normalizing *H*.

One of the basic measures is to normalize with respect to the maximum value *H*_max_ = log *n* which is the signature of a uniform distribution. The first measure is the entropy ratio *r* defined as

6 $$ r = \frac{H}{{H_{\hbox{max} } }} = \frac{{ - \sum\nolimits_{i} {p_{i} \,\log p_{i} } }}{\log n}, $$

which we have worked out for the three-event system in Fig. 1 as 0.748. In fact, the complement of this redundancy *R* is the more usual measure. We call *R* the complexity ratio defined as *R* = 1 − *r* which can be written as

7 $$ R = 1 - \frac{H}{{H_{\hbox{max} } }} = \frac{{H_{\hbox{max} } - H}}{{H_{\hbox{max} } }} = \frac{I}{{H_{\hbox{max} } }}, $$

which is the percentage of information that the system could realize by adjusting itself to the most probable state, that is, a uniform distribution. As such, this might be a measure of ‘slack’ in the system. The key to relative information lies in the difference *H*_max_ − *H* which in a much more direct form is the classic information difference *I* (see Kullback and Leibler 1951; Kullback 1959; Theil 1972). This can be written as

8 $$ \begin{aligned} I & = H_{\hbox{max} } - H = - \sum\limits_{i} {p_{i} \,\log \left( \frac{1}{n} \right)} + \sum\limits_{i} {p_{i} } \,\log p_{i} \\ & = \sum\limits_{i} {p_{i} \,\log \left( {\frac{{p_{i} }}{1/n}} \right)} . \\ \end{aligned} $$

If we assume that the prior probability is

9 $$ q_{i} = 1/n,\quad \sum\limits_{i} {q_{i} = 1} , $$

then Eq. (8) can be written as a generic information difference measure in its classic form as

10 $$ I = \sum\limits_{i} {p_{i} \,\log \left( {\frac{{p_{i} }}{{q_{i} }}} \right)} . $$

It is easy to see that this difference provides a clearer focus on the role of the number of events or zones. However, this tends to discount the effect for as a system changes through the addition of more events, information difference formulas simply measure relative change. We need to return to absolute differences in the quest to disentangle the density of the distribution from the number of events, and to this end, we now need to deal directly with probability densities.

### Spatial information

What we have not done so far is consider the size of the event with respect to its probability. Generally in a spatial system, if the area over which the probability is measured increases, the density will change. To incorporate this effect explicitly in the definition of information, we need to define an approximation to the density over an area Δ*x*_*i*_ where the total area of the system *X* is

11 $$ \sum _{i}\Delta x_{i} = X. $$

The density can be approximated as

12 $$ p(x_{i} ) = \frac{{p_{i} }}{{\Delta x_{i} }},{\text{ or}}\,\,\,p_{i} = p\left( {x_{i} } \right)\Delta x_{i} , $$

and we will assume that in the limit, Eq. (12) converges to the probability density *p*(*x*) which we define for completeness as

13 $$ p(x) = \mathop {\lim }\limits_{{\varDelta x_{i} \to 0}} \frac{{p_{i} }}{{\Delta x_{i} }}. $$

We can now write the entropy formula *H* in probability density terms as

14 $$ \begin{aligned} H & = - \sum\limits_{i} {p(x_{i} )\Delta x_{i} \,\log (p(x_{i} )\Delta x_{i} )} \\ & = - \sum\limits_{i} {p(x_{i} )\,\log } (p(x_{i} ))\Delta x_{i} - \sum\limits_{i} {p(x_{i} )\,\log } (\Delta x_{i} )\Delta x_{i} . \\ \end{aligned} $$

If we now pass to the limit, then the first term on the right-hand side of Eq. (14) converges to the continuous form of entropy *S* while the second term diverges to infinity due the fact that the number of events *n* must diverge in this way.^2^ We can then write Eq. (14) as

15 $$ \begin{aligned} \mathop {\lim }\limits_{{\Delta x_{i} \to 0}} H & = - \int_{x} {p(x)\,\log p(x){\text{d}}x - \int_{x} {p(x)\,\log ({\text{d}}x){\text{d}}x} } \\ & = S - \int_{x} {p(x)\,\log ({\text{d}}x){\text{d}}x} . \\ \end{aligned} $$

We will now define the discrete entropy in terms of the distribution and the area of each event as

16 $$ \begin{aligned} H & = - \sum\limits_{i} {p_{i} \,\log \frac{{p_{i} }}{{\Delta x_{i} }}} - \sum\limits_{i} {p_{i} \log } \,\Delta x_{i} \\ & = S + Z, \\ \end{aligned} $$

where *S* is the approximation to the continuous entropy and *Z* is the approximation to the information associated with the sizes of the events comprising the distribution which enable densities to be measured. *S* is the formula that we called ‘spatial entropy’ in our earlier work (Batty 1974), but here we focus on *H* which is composed of this term and the information density *Z*. In short, when we examine *H*, we will do this with respect to the numerical co-variation of its elements *S* and *Z*.

### Varying spatial information

We will examine the range from the minimum to the maximum values of spatial entropy and information density in Eq. (16), but before we do so, it is worth noting that the limits on *H* are exactly the same as those noted above with *H*_min_ = 0 and *H*_max_ = log *n*. This is easy to show for the term *Z* factors out from Eq. (16) leaving the basic Shannon formula in Eq. (4). We will now show the limits for *S* and *Z*, noting that the distribution *p*_*i*_ can take on two extreme forms

17 $$ p_{k} = \left\{ {\begin{array}{*{20}l} {1,} \hfill & {i = k} \hfill \\ {0,} \hfill & {\text{otherwise}} \hfill \\ \end{array} } \right.\quad {\text{or}}\quad p_{i} = \frac{1}{n}\;,\quad \forall i, $$

and

18 $$ \Delta x_{k} = \left\{ {\begin{array}{*{20}l} {X,} \hfill & {i = k} \hfill \\ {0,} \hfill & {\text{otherwise}} \hfill \\ \end{array} } \right.\quad {\text{or}}\quad\Delta x_{i} = \frac{X}{n}\;,\quad \forall i, $$

In fact, we will assume that when only one zone or event has a size associated with it as in Eq. (18), then this event is that associated with the single probability in Eq. (17); that is, the event *k* is the same for the first term in Eq. (17) and the first in (18).

Shannon’s entropy is dimensioned with respect to the number of events *n* although its components, spatial entropy and the information density, depend on the measure *X*. If we normalize the measure to unity, then it is clear from Eq. (11) that the distribution of event sizes can be considered a probability distribution

19 $$ q_{i} = \sum\limits_{i} {\Delta x_{i} = 1} . $$

Spatial entropy is now the negative of the information difference, that is,

20 $$ S(x) = - \sum\limits_{i} {p_{i} \,\log \frac{{p_{i} }}{{q_{i} }}} = - I $$

and the information density is the expected value of this event size distribution with respect to the population distribution, that is,

21 $$ Z\left( x \right) = - \sum\limits_{i} {p_{i} \, { \log } q_{i} } . $$

This means that our analysis can be considered one of examining information differences, but it is essential that we take account of the measure *X* when we are involved in interpreting the two components of the traditional Shannon entropy.

Our first foray into measuring how the information we are assuming is a proxy for complexity as cities become larger and as their distributions of population change, follows this section. But before we present this, it is worth noting the range of values for entropy and its two components using the extremes of the two distributions indicated in Eqs. (17) and (18). Using these definitions, we show the limits for *H*, *S* and *Z* in Table 1 where it is clear that the spatial entropy term has a maximum of log *X* and a minimum of log *X* − log *n*. The information density always cancels this term to provide the extreme value of *H* which is shown in the far right-hand column of the table.

**Table 1:** Extreme values of entropy statistics

Range of *p*_*i*_ and Δ*x*_*i*_	*S*	*Z*	*H*
$$ \begin{gathered} p_{i} = 1/n \hfill \\\Delta x_{i} = X/n \hfill \\ \end{gathered} $$	log *X*	log *X* − log *n*	log *n*
$$ \begin{gathered} p_{i} = 1/n \hfill \\\Delta x_{i} = X,\;0\,\;{\text{for}}\;{\text{one}}\;i \hfill \\ \end{gathered} $$	$\log n + \frac{\log X}{n}$	$- \frac{\log X}{n}$	log *n*
$$ \begin{gathered} p_{i} = 1,\;0\,\;{\text{for}}\;{\text{one}}\;i \hfill \\\Delta x_{i} = X/n \hfill \\ \end{gathered} $$	log *X* − log *n*	−log *X* + log *n*	0
$$ \begin{gathered} p_{i} = 1,\;0\,\;{\text{for}}\;{\text{one}}\;i \hfill \\\Delta x_{i} = X,\;0\,\;{\text{for}}\;{\text{one}}\;i \hfill \\ \end{gathered} $$	log *X*	− log *X*	0

There is another measure that is useful for examining changes in the number of events and their density. If we focus on the distribution *q*_*i*_ as defined from Eq. (19) which normalizes the distribution of land area to sum to unity, then we can form the expected value of its logarithm defined as *H*(*p*). We can compare this to the entropy of the distribution itself *H*(*q*) where these measures are defined as

22 $$ H\left( p \right) = - \sum\limits_{i} {p_{i}  \,{ \log } q_{i} } \quad {\text{and}} $$

23 $$ H\left( q \right) = - \sum\limits_{i} {q_{i}  \,{ \log } q_{i} } . $$

The first measure *H*(*p*) gives the evenness of the density distribution with respect to the population while the second provides the same with respect to the density. Comparing these two terms, this is a measure of difference posed by the way the area of zones or sizes of the events are represented. Tribus and McIrvine (1971) introduced a composite measure based on the sum of the two measures defined as *H*(*p*/*q*) which is

24 $$ \begin{aligned} H(p/q) & = H(q) - H(p) \\ & = \sum\limits_{i} {p_{i} \,\log q_{i} - \sum\limits_{i} {q_{i} \,\log q_{i} } } . \\ \end{aligned} $$

Of course, the information difference in Eq. (10) measures a similar difference which can be written as

25 $$ I = \sum\limits_{i} {p_{i} \,\log p_{i} - \sum\limits_{i} {p_{i} \,\log q_{i} } } $$

and we would expect these two measures in Eqs. (24) and (25) to co-vary. The difference in absolute terms becomes greater the greater the difference between the distributions of population and of land area or size. We now have a large enough arsenal of tools to explore real problems, albeit in their simplest form. To this end, we elaborate four examples of how the number of zones *n* and the density of population *p*_*i*_/Δ*x*_*i*_ trade-off against each other in computing complexity based on information using data from population in the London metropolitan region.

## Preliminary examples: changes in density and number of zones

Our first demonstration is based on the changing dynamics of the population distribution in 33 London boroughs over the last 100 years. In fact, the population of the metropolis is fairly stable over this period starting at some 6.5 million in 1901, reaching 8.1 m in 1951, falling back to 6.5 m and climbing back to 6.7 m in 2001. Recently, it reached 8.1 m in 2011. We keep the number of boroughs the same, and there are no boundary changes during this period from 1901 to 2001 for we have standardised the boundaries to 2001, so our examination is entirely focused on the density of population. In fact, the density changes from the lowest in 1901 and reflects small oscillations in the distribution over the 100-year period. Our general intuition about the metropolitan area is that population has decentralized over this period with outer London boroughs gaining in population and central boroughs losing population. We might therefore expect the information entropy to be increasing due to the flattening of the population density surface but as we will see, this is not quite so straightforward.

**Fig. 3:**
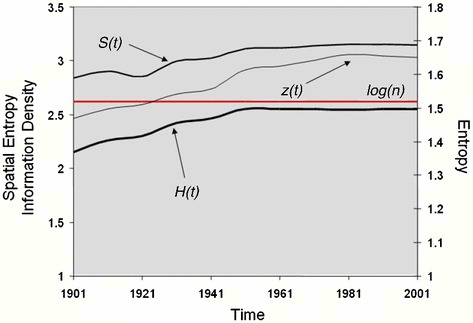
Entropy distributions from 1901 to 2001

In Fig. 3, we show the entropy *H* and its two components *S* and *Z* where we now notate these variables with respect to the time *t* at which they are measured. Each of these measures increases in value with the Shannon entropy approaching its maximum towards the end of the period, largely because the London boroughs have acquired more equal populations through time. We also note that spatial entropy and information density are measured in terms of square kilometres (*X* = 1,594.5 km^2^). However, there are some ‘tiny’ reversals in values over this period with entropy itself falling in value—the city itself ‘losing complexity’, from 1961 to 1981 when population was falling most rapidly during a period of massive suburbanization outside the metro area and during a period of intense deindustrialization. In the last 20 years, population has been returning to the city and international migration has added to the mix.

**Fig. 4:**
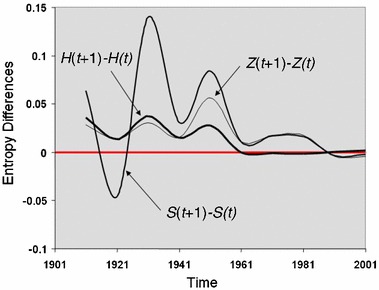
Entropy differences from 1901 to 2001

We show these reversals in terms of the three measures *H*, *S* and *Z* in Fig. 4, where it is clear that there are some large shifts in spatial entropy and information density with this value falling in the 1920s, then reversing in the 1930s and war years, then falling in the post-war years, rising in the 1950s and 1960s, and then becoming more stable since that time. These values are hard to interpret because as yet we do not have strong links to density distributions to which they are clearly linked. However, when we examine the density itself, then the falls in density occur in the periods 1931–1941 and 1951–1981 with a slow rise in density since then. In fact, we know from other evidence that the metropolis is still losing its indigenous population, the difference being made up by a more transient population which is coming for education or work mainly from the European Union which in the last decade has essentially become a free labour market.

**Fig. 5:**
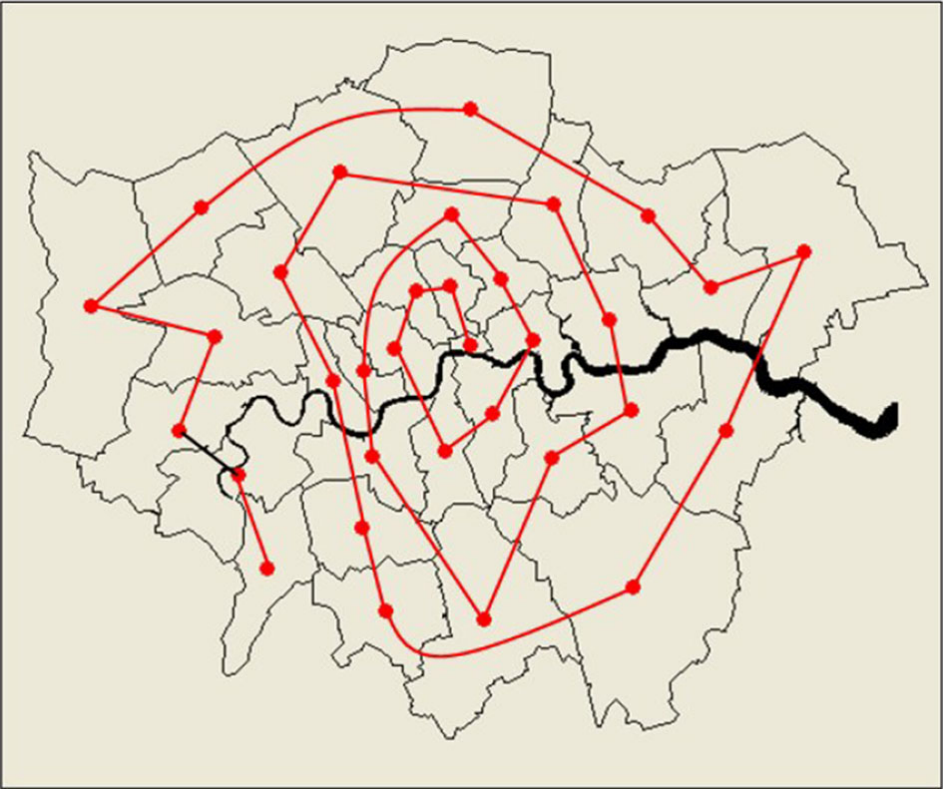
Spatial aggregation of zones according to a spiral from the two most central to the outer boroughs

Before we look at the wider London region where we have much larger changes in the number of zones and the density of population, our second example will examine what happens if we simply increase the number of zones *n* from a minimum of 2 to all 33, adding borough by borough but following a concentric spiral emanating from the core of the city—the City of London and its neighbour Islington and then winding around taking in boroughs which are further and further out. Thus, we have an increase in complexity due to the number *n* which from our previous analysis appears to be more significant than distribution in terms of these examples. However, in London, population densities are low in the centre and rise in the inner areas to fall again as one approaches the outer suburbs. The spiral traced out is shown in Fig. 5 where we have simply taken zones that are adjacent. We have not used any criterion other than adjacency to construct this aggregation, but the spiral does trace out the pattern of density that has emerged as the city has grown out from its historic core.

**Fig. 6:**
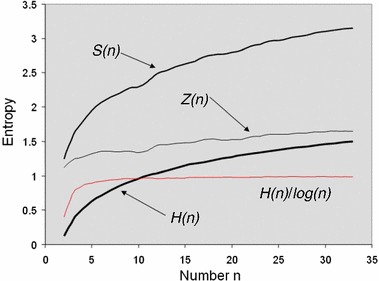
Entropy statistics associated with the spiral aggregation

The changes in entropy statistics—Shannon and its decomposition into spatial entropy and information density—are plotted in Fig. 6, and as we can see, there is a substantial change. In fact if we were to plot these statistics against log *n* rather than *n*, entropy *H* is almost linear revealing that despite some marginal changes in density, the number of zones is by far the largest determinant of the level of complexity. To an extent this is what we might expect for this example for the population distribution is for one time only—2001 is the distribution that we have used here—and we would not expect there to be big reversals in the density profile over space rather than time. In fact, there are very few reversals in value for any of the three statistics with the exception of the information density *Z*(*x*) which does not appear of any consequence and is probably due to local factors. As one might expect, with small numbers of zones, the changes in entropy are proportionately larger as one might expect by simply reflecting on changes in the maximum entropies (log *n* + 1 − log *n*)/log *n* which get smaller as *n* increases.

**Fig. 7:**
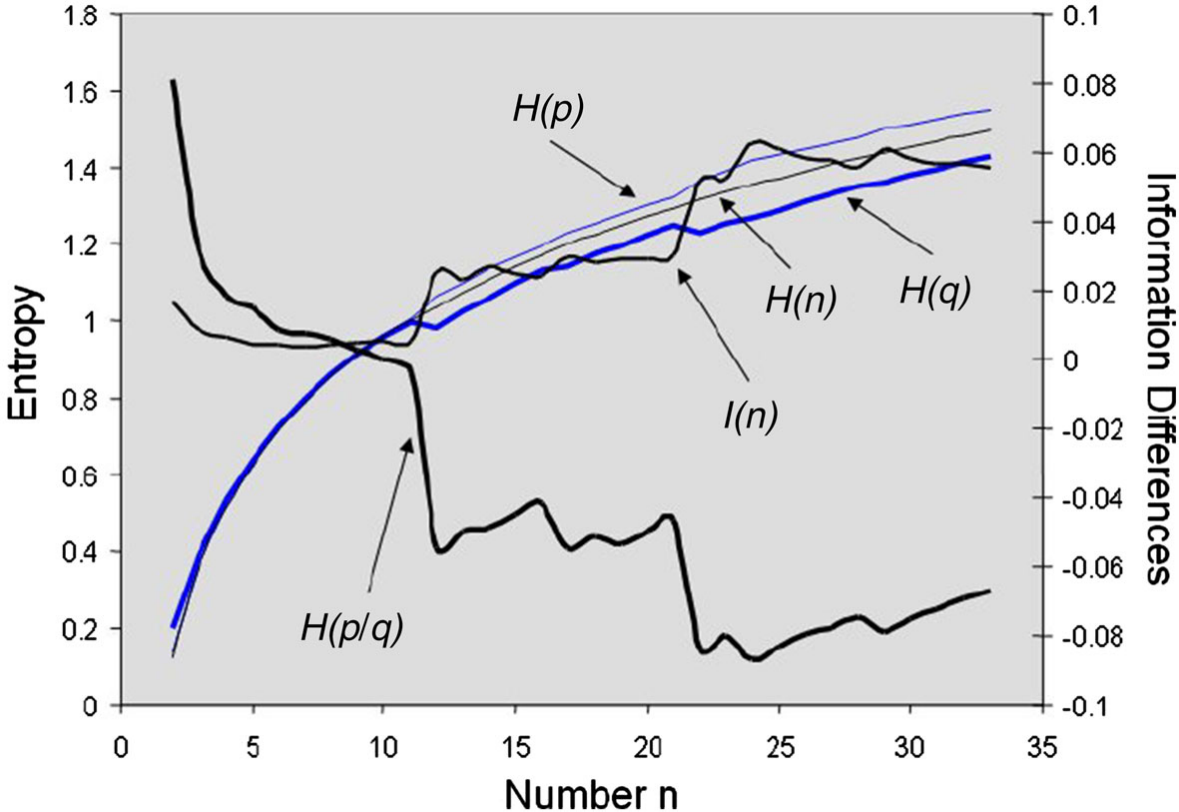
Entropy and information differences associated with the spiral aggregation

If we now plot the information difference, the Tribus and McIrvine (1971) information difference measures and their components in Eqs. (21)–(25), we see the key differences between the population and land area distributions. The entropy of the population distribution *H* lies between the entropy *H*(*q*) and the entropy *H*(*p*), and this gets greater as the number of zones increases. This implies that the information difference also gets greater which means the two distributions—population and land—become more unlike as we move further away from the core of the metropolis. In fact, population densities fall, but this can be accounted for by an increase in area and decreases in population or any combination thereof. From these statistics, it is not easy to figure this out. We show these differences in Fig. 7.

## Changes in density and zones defining a large metropolitan region

**Fig. 8:**
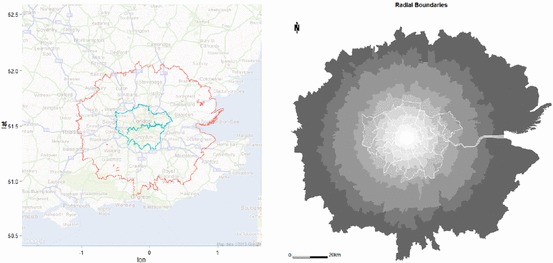
The complete metropolitan region, organized into distance bands

Our third major demonstration deals with a much larger system where we might expect to see much greater variations in information. We have defined a system of 1,767 zones of which the Greater London Authority area is now an aggregated set of zones comprising the inner core as we show in Fig. 8. This region is approximately the Inner and Outer Metropolitan regions (less a small area near the coast), and the zones in this case are administrative wards (local electoral districts) which have an average of 7,600 persons per zone. The region in question has a population of 13,428,850, its area is 13,004 km^2^, and its average density is 1,033 persons/km^2^ varying over a range from a maximum of 20,794 persons/km^2^ to a minimum of 32 persons/km^2^. We have organized the zones by distance from central London (Charing Cross) to give nine bands, and these are shaded in Fig. 8 to match those in the subsequent figures so as to provide some sense of location.

**Fig. 9:**
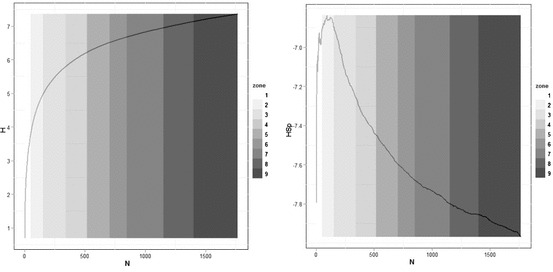
*Left* Entropy *H* and *right* spatial entropy *S*

We have plotted the entropy *H* and the spatial entropy *S*(*x*) in Fig. 9. As expected, the entropy rises linearly in approximate proportion to its maximum value log *n*. The spatial entropy is much more sensitive, and as this is an information difference, it rises rapidly at first but after about 100 zones falls. To an extent, this can be interpreted as the fact that in the vicinity of the historic core, the population is relatively low density but it then reaches a high in the inner areas of London, only to fall off quite rapidly as the Greater London Authority boundary is reached at around 500 zones.

**Fig. 10:**
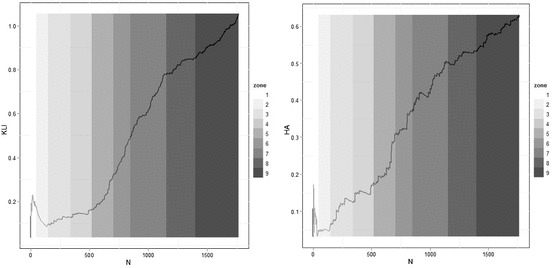
*Left* Information *I* and *right* Tribus–McIrvine *H*(*p*/*q*)

As we know that the maximum value of entropy is log *n* and the maximum value of spatial entropy is log *X*, the difference between them is the maximum information density. In fact, this difference is picked up to an extent in the Kullback–Leibler and Tribus and McIrvine information statistics. We show these in Fig. 10 where it is quite clear that these show a marked degree of variation around the initial 100 zones although this might be a reflection of random variation across relatively small numbers of zones. This suggests that the variation is a spatial effect reflecting density variations in the historic core which we noted above. The drop in value of Kullback–Leibler information across the first and second zones reflects the increasing dominance of lower population figures in larger zones. This effect is less marked in the Tribus–McIrvine case suggesting a damping effect arising from the additional area term {*q*_*i*_}.

**Fig. 11:**
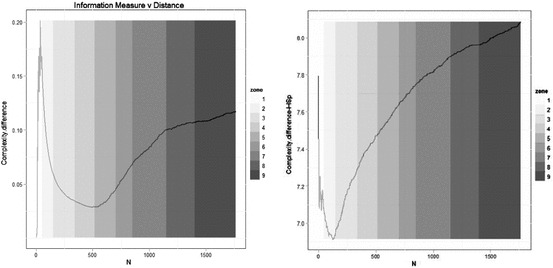
The information difference *H*_max_—*H* and the information difference minus spatial entropy

To summarize these measures, we will plot the complexity difference *RH*_max_ = *H*_max_ − *H* which is the information with the prior set as the maximum entropy or uniform distribution. We noted earlier that this is a version of the Kullback–Leibler information statistic and it is the amount of information required to change the existing distribution to its maximum entropy which is the uniform distribution. If we use the ratio, then this is the information required in percentage terms, but in Fig. 11 we plot *H*_max_ − *H*.

*H*_max_ represents the maximum possible information contained in the distribution and *H* the actual level of information so *H*_max_ − *H* is a measure of our ignorance and it thus reflects the degree of complexity in the system under consideration. This difference is related to spatial entropy in Fig. 11 where the information difference minus the spatial entropy is plotted. It shows that the spatial entropy sharpens the turning points. At the core of the city, the difference is very small but it rises dramatically showing that high-density population dominates the inner areas. Then, as the city spreads out the difference between a uniform and the actual distribution gets less, but after 500 zones the differences become much greater showing an increase in overall density. All of these differences are masked by the overall entropy statistic which rises inexorably as Fig. 9 suggests. However, if we plot entropy against log *n*, the increase is linear, thus suggesting that apart from some very minor oscillations, added information produced by adding to the number of zones far outweighs any differences in complexity generated by the changing density of population.

## Spatial growth in population infrastructure over long historical periods

Our last example at first sight provides much greater possibilities for a reversal in complexity as the city grows in terms of zones. We have used a very detailed database of street intersections which we have aggregated to 400 m × 400 m grid squares for the Greater London region. As we have the evolution of the street system since 1786, we impose a density threshold and count all intersections greater than this threshold for all nine time periods from 1786 to 2010 (Masucci et al. 2013). The density threshold defines those grid squares that comprise the city, and the count of intersections above this threshold defines the density of each grid square. This gives us a population of the density of street intersections for each cross-section. As we might expect, the number of cells increases through time as the city expands and more and more grid squares with streets within them that are above the density threshold emerge. The number of grid squares goes from 123 in 1786 to 6,952 in 2010 with the progression being logistic in that the rate of change has considerably reduced in the last 20 years as the city system nears its developed capacity. This capacity is to a large extent determined by the hard green belt that has limited growth of the metropolis to within the Greater London boundary. The maximum density in 1786 was 40 intersections, and by 2010, it had only reached 43 which shows that the system reaches its maximum density very quickly as the street system evolves.

**Fig. 12:**
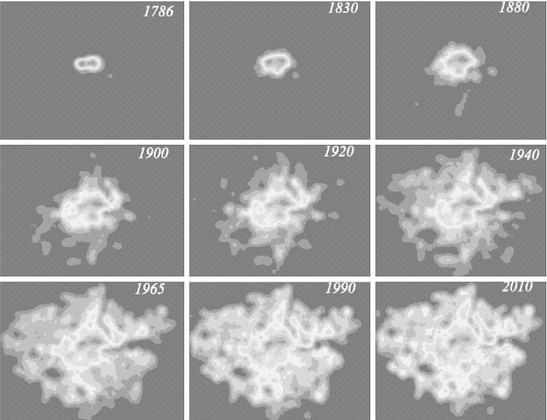
The density of street intersections in Greater London 1786–2010

**Fig. 13:**
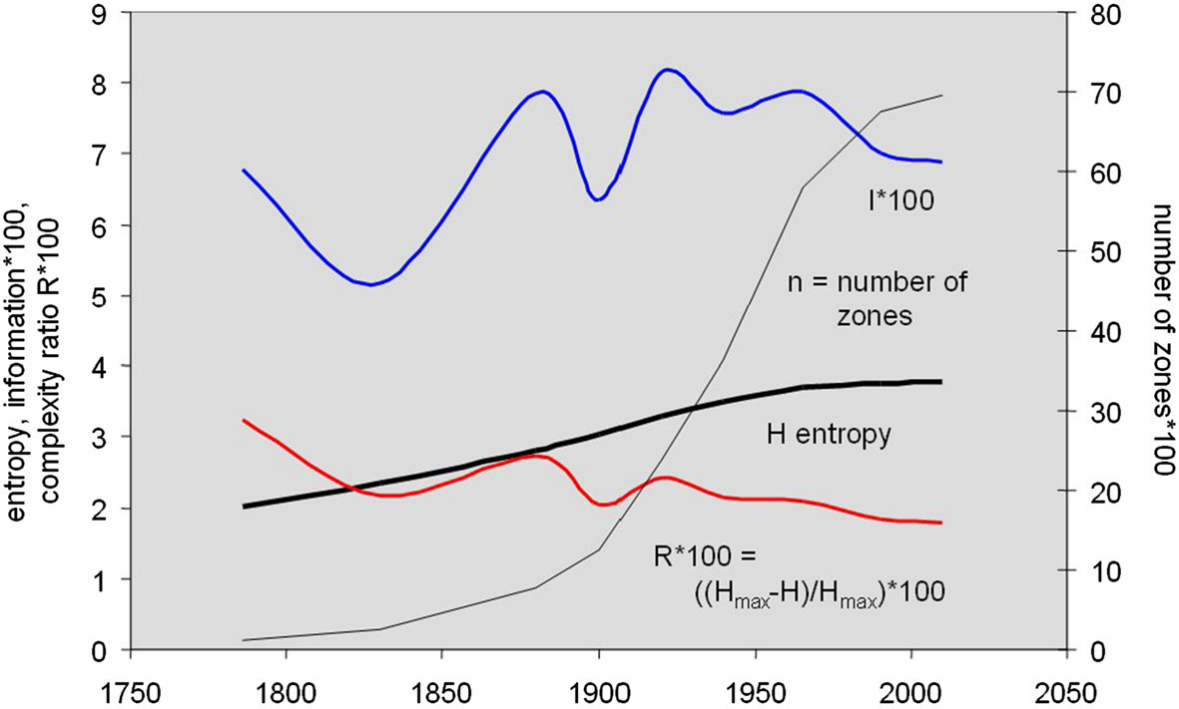
Changes in information and complexity in the population of streets

Like the development of population in Greater London, the development of the street pattern follows a logistic evolution with its capacity being approached within the last 20 years. We show the development of the city in terms of these densities in Fig. 12, and it is clear that the number of zones is likely to dominate the entropy rather than the density of the street patterns which move towards an upper limit of a maximum of between 40 and 50 per zone as the system fills up. In Fig. 13, we show the growth in the number of zones which is likely to dominate the change in information, notwithstanding the changes in density that lead to a more uniform density through time. We have computed all the measures that we have used previously, but the spatial entropy measure is less useful because the data are already in density form; that is, the distribution of probabilities is a density distribution because each area is identical in size, that is, 0.4^2^ = 0.16 km^2^. In this sense, the entropy that can be partitioned into its spatial and information density components *H* = *S* + *Z* means that *S* varies as the difference between *H* and log *X*, the land area of the system. In fact, we will not plot the spatial entropy but focus on the entropy itself, the complexity ratio as a percentage, and the information measure.

Figure 13 shows that the entropy rises more rapidly at first and begins to stabilize as the system approaches capacity—this is implicit too in the logistic growth in the number of zones above the basic density threshold. In fact, the percentage change in entropy from time period to time period goes from around 0.3–0.4 % per annum between 1786 and 1920 and then falls systematically through most of the twentieth century to 0.02 % per annum from 1990 to 2010. In fact, this implies that the system has almost reached capacity and that it is unlikely that it can grow much more, at least within the Greater London area that is the inner core of the wider region (Masucci et al. 2013). If we examine the complexity ratio *R*, which reveals the percentage of information needed at any time period to move the system to its maximum complexity, this falls slowly but systematically and reaches <2 % by the end year of the analysis. This small value shows how significant the effect is of the number of zones on the various complexity measures, and it might be argued that we need to weight the effect of density more significantly and reduce the impact of the number of events in terms of this measure. This is also reflected in the value of the Kullback–Leibler information statistics which correlates with the complexity ratio as illustrated in Fig. 13.

It is perhaps a little disappointing that in most of our examples we do not get the kinds of reversals in complexity or information that we were seeking although it is necessary to note that importance of the number of events in these formulas. As we have just argued, we probably need to develop our measures of complexity to the point where the trade-off between numbers of zones and the spread of population is handled more evenly than that is implied in the use of Shannon’s formula. What this also illustrates is that it is most important to choose examples carefully. In some respects those chosen in this paper are as severe a test of these ideas as any because over the space–time series used for the growth of London, it might be expected that in general, the city has become ever more complex as it has grown. Nevertheless, we do see a complex trade-off between density and the number of events, and this suggests that there is considerable work still to do on unravelling these measures and linking them to other measures of diversity.

## Next steps: an emerging research agenda

The obvious extension of these measures is to two-dimensional spatial systems which focus on interactions. Formal extensions are straightforward but interpretations are complex, and there are many configurational issues relating to the density of origins and destinations and their entropies that relate to the interaction entropy. There are many applications of joint information measures that relate directly to these extensions, but the whole question of trading-off resolution in terms of densities of interactions against number of interactions is quite complicated in terms of the analysis we have developed here. This poses an important challenge to be addressed in future work.

The bigger issue is how these information measures extend to other types and definitions of complexity, first with respect to information and entropy and then with respect to measures of diversity. There has been a long but rather fruitless effort so far in terms of defining complexity, much of this work based on information theory but the verdict is still out with respect to the usefulness of this approach (Gell-Mann and Lloyd 1996; Gershenson and Fernandez 2012). It is plagued with definitional, relational and interpretational pitfalls some of them discussed in this paper but these might fruitfully be addressed by the use of more generalized entropies proposed by Thurner (2007) and Tsallis (2009). Moreover, the need for good examples is paramount, and it is important in future work to explore examples where there is clear and incontrovertible evidence of cities getting less complex as they evolve. For the most part, this is not the case although over much longer historical periods, there is much casual evidence that this is the case. In fact, it is likely that cities evolve in fits and starts and there is some evidence in our examples here that this is the case although reversals in complexity are hard to find in our data. Nevertheless, cities reach a threshold in which technologies are consolidated, almost as though they await the next wave of change.

In progressing these ideas, there is an urgent need for better examples, for more varied cases and for the extension of these ideas to different kinds of population—other than streets and people. If the range of populations was broadened, then it might be possible to disaggregate these measures further to deal with coupled populations, thus directing research onto much more diverse systems. This is a challenge that we will take up in future papers.

## Footnotes

1 We assume here, and this is controversial, that if an event has zero probability of occurring, then the event is still intact and the relevant component of the $H$ formula is defined as $0\log (0) = 0$. There is an argument for saying that if the event has zero probability, the event does not exist and the system must be redefined to exclude the event in question, hence reducing its dimensionality.

2 This is an informal result of the limiting process which is considerably more tricky to demonstrate if the coordinate system and its measure are taken into account as Jaynes (1963, 1968) has noted. In fact as all our work here is empirical, we do not deal with these limits *per se* and it is the discrete formulation that is important. We will see that this form is relatively well behaved and does not pose any major difficulties (for a more detailed note, see Batty 1974).
